# Risk‐Treatment Paradox in the Selection of Transradial Access for Percutaneous Coronary Intervention

**DOI:** 10.1161/JAHA.113.000174

**Published:** 2013-06-21

**Authors:** Neil J. Wimmer, Frederic S. Resnic, Laura Mauri, Michael E. Matheny, Thomas C. Piemonte, Eugene Pomerantsev, Kalon K. L. Ho, Susan L. Robbins, Howard M. Waldman, Robert W. Yeh

**Affiliations:** 1 Division of Cardiovascular Medicine, Brigham and Women's Hospital, Harvard Medical School, Boston, MA (N.J.W., L.M.); 2 Department of Cardiovascular Medicine, Lahey Clinic, Burlington, MA (F.S.R., T.C.P., S.L.R.); 3 Tufts University School of Medicine, Boston, MA (F.S.R., T.C.P., S.L.R.); 4 Department of Medicine, Vanderbilt University Medical Center, Nashville, TN (M.E.M.); 5 Cardiology Division, Massachusetts General Hospital, Harvard Medical School, Boston, MA (E.P., R.W.Y.); 6 North Shore Medical Center, Salem, MA (H.M.W.); 7 Beth Israel Deaconess Medical Center, Harvard Medical School, Boston, MA (K.L.H.)

**Keywords:** catheterization, complications, coronary disease, stents

## Abstract

**Background:**

Access site complications contribute to morbidity and mortality during percutaneous coronary intervention (PCI). Transradial arterial access significantly lowers the risk of access site complications compared to transfemoral arteriotomy. We sought to develop a prediction model for access site complications in patients undergoing PCI with femoral arteriotomy, and assess whether transradial access was selectively used in patients at high risk for complications.

**Methods and Results:**

We analyzed 17 509 patients who underwent PCI without circulatory support from 2008 to 2011 at 5 institutions. Transradial arterial access was used in 17.8% of patients. In those who underwent transfemoral access, 177 (1.2%) patients had access site complications. Using preprocedural clinical and demographic data, a prediction model for femoral arteriotomy complications was generated. The variables retained in the model included: elevated age (*P*<0.001), female gender (*P*<0.001), elevated troponin (*P*<0.001), decreased renal function or dialysis (*P*=0.002), emergent PCI (*P*=0.01), prior PCI (*P*=0.005), diabetes (*P*=0.008), and peripheral artery disease (*P*=0.003). The model showed moderate discrimination (optimism‐adjusted c‐statistic=0.72) and was internally validated via bootstrap resampling. Patients with higher predicted risk of complications via transfemoral access were less likely to receive transradial access (*P*<0.001). Similar results were seen in patients presenting with and without ST‐segment myocardial infarction and when adjusting for individual physician operator.

**Conclusions:**

We generated and validated a model for transfemoral access site complications during PCI. Paradoxically, patients most likely to develop access site complications from transfemoral access, and therefore benefit from transradial access, were the least likely to receive transradial access.

## Introduction

Bleeding and other access site complications are significant contributors to the morbidity and mortality associated with percutaneous coronary intervention (PCI).^1–9^ The use of transradial arterial access significantly lowers, but does not eliminate, the risk of bleeding and other vascular complications compared with the transfemoral approach.^10–16^ However, across the United States, the rate of transradial arterial access remains low. In an analysis of more than 1.7 million hospital admissions for PCI from the National Cardiovascular Data Registry (NCDR) CathPCI database from 2005 to 2009, the rate of transradial arterial access for PCI was <1.5%.^17^ As operator enthusiasm and experience with transradial access grow, the utilization of this approach is likely to increase significantly over the next several years.

As with other interventions and therapies, the magnitude of benefit associated with transradial access is likely to vary widely among patients, depending primarily on patient risk of femoral vascular access complications. As transradial arterial access for PCI becomes more widely adopted, ensuring that it is used preferentially in patients at highest risk of vascular access complications is essential for optimizing patient outcomes and avoiding the pitfalls of the “risk‐treatment paradox”—the phenomenon that interventions are often least used in patients expected to benefit the most from them.^18^ However, tools to enable clinicians to stratify patients on the basis of their risk for access‐site complications are currently limited.

The purpose of this analysis was 2‐fold. First, we sought to develop a prediction model for access site complications using preprocedural clinical and demographic factors in patients undergoing PCI via transfemoral arterial access in the current era. Second, we sought to assess whether transradial arterial access was rationally applied as a complication avoidance strategy by being performed in patients at the highest risk for complications via the transfemoral approach.

## Methods

### Study Population

Data were obtained on consecutive patients who underwent PCI without intra‐aortic balloon pump or other mechanical circulatory support from January 1, 2008 through December 31, 2011 at 5 institutions in Massachusetts using either transfemoral or transradial arterial access. The institutions included Brigham and Women's Hospital, Massachusetts General Hospital, Beth Israel Deaconess Medical Center, the Lahey Clinic, and North Shore Medical Center. Deidentified, patient‐level data were extracted using the NCDR CathPCI Registry forms versions 3 or 4 as part of the Data Extraction and Longitudinal Trend Analysis (DELTA) network study.^19^ PCI was performed according to standard clinical practice.

### Definitions

The dataset is based on the American College of Cardiology—National Cardiovascular Data Registry definitions and contains clinical and procedural elements for each patient and follow‐up information for the occurrence of all in‐hospital complications.^20–21^ A full description of the data element definitions for the CathPCI registry is available online at http://www.ncdr.com/WebNCDR/ELEMENTS.ASPX.

The outcome of interest was vascular access site complications, which were defined as one of the following occurring during the index hospitalization: access site bleeding requiring transfusion, access site bleeding causing hematoma >5 cm, retroperitoneal bleeding, other vascular complications requiring diagnostic testing or therapy (for instance, pseudoaneurysm), or death from a vascular cause.

We considered a range of candidate predictors for possible inclusion in the prediction model based on clinical relevance. These factors included demographic information, factors related to the general medical history (including body mass index), factors related to the cardiovascular history, factors related to the acute presentation (including the presence of clinical shock, whether the PCI was emergent, the presence of ST‐segment elevation myocardial infarction [STEMI], current renal function, etc), and factors known to be associated with PCI‐related bleeding or mortality.^22–23^

### Statistical Approach

Continuous variables were described as means (±standard deviation) and were compared using *t*‐tests due to large sample size. Categorical variables were described as percentages and compared using chi‐square or Fisher exact tests. We modeled in‐hospital vascular access site complications among patients who had transfemoral arterial access using multivariable logistic regression methods. Models were generated using backward selection with univariate prescreening of candidate predictors (*P*<0.2 for entry, *P*<0.05 for retention). After univariate screening, 15 variables were considered for retention in the final model. These variables included: age, female sex, prior congestive heart failure, history of diabetes mellitus, history of chronic lung disease, history of hypertension, history of peripheral artery disease, chronic kidney disease (glomerular filtration rate ≥60 mL min^−1^ 1.73 m^2^, <60 mL min^−1^ 1.73 m^2^, or on chronic dialysis), history of prior valvular heart surgery, whether the procedure was emergent, the presence of an elevated troponin (either troponin T or troponin I) prior to the procedure, presentation with STEMI, or presentation with non‐ST segment myocardial infarction.

Model discrimination was assessed with the c‐statistic. Model calibration was assessed by plotting a smoothed line of observed versus predicted probabilities of vascular complications within the femoral PCI population, as well as via the Hosmer–Lemeshow chi‐square test.^24^ We internally validated the model in 1000 bootstrap samples (resampling with replacement), as this method has been shown to perform more reliably than other methods of internal validation.^25–26^ Final reported estimates of model discrimination and calibration were reduced to account for optimism based on previously described methods.^25^ Model coefficients were adjusted based on a calculation of the linear calibration slope in order to adjust for overfitting.^25,27^

The prediction model for femoral access site complications was then applied to all patients in the study sample, including those who received PCI via a transradial approach. Patients in both the transfemoral and transradial groups were stratified into risk categories of <1% risk of complications, 1% to 2% risk of complications, and >2% risk of complications. Comparisons of the percentage of patients receiving transradial access compared to transfemoral access were then made across risk groups. Similar analyses were performed in individuals who presented with STEMI, those who did not present with STEMI, as well as in the overall population excluding patients receiving dialysis treatment. We additionally quantified the relationship between risk of vascular complications with a transfemoral approach and the actual receipt of transradial access using logistic regression.

To account for the individual physician operator in the relationship between the risk of transfemoral vascular access site complications and the use of transradial access, we generated a 2‐level mixed effects logistic regression model with a random effect for the physician operator. Patients were included in this model if the physician performing the PCI had performed at least 25 procedures in the time under study. Individual physician operator information was available from 4 of the 5 clinical sites.

Analyses were performed using STATA 11.2 (Statacorp) and R (version 2.15.0). The study was Institutional Review Board approved by the Partners Human Research Committee.

## Results

A total of 17 509 patients undergoing PCI met the inclusion criteria for this analysis. Transfemoral arterial access was used in 14 387 (82.2%) patients and transradial arterial access was used in 3122 (17.8%) patients. Baseline characteristics overall, and stratified by arterial access site, are shown in Table 1.

**Table 1.** Baseline Characteristics of Patients Undergoing PCI Via the Transfemoral or Transradial Approach

Variable	Total (n=17 509)	Femoral (n=14 387)	Radial (n=3122)	*P* Value
Age (standard deviation), y	65.9 (12.2)	66.3 (12.2)	64.2 (11.8)	<0.001
Male, %	71.5	70.7	75.3	<0.001
Black, %	3.6	3.5	4.3	0.038
Prior myocardial infarction, %	32.9	33.5	30.2	<0.001
Prior heart failure, %	12.0	13.2	8.2	<0.001
Diabetes, %	32.7	33.9	32.1	0.057
Chronic lung disease, %	13.6	13.7	13.4	0.62
Prior PCI, %	35.8	37.5	32.9	<0.001
Prior CABG, %	17.4	19.7	9.5	<0.001
Hypertension, %	82.0	82.2	81.3	0.212
Peripheral artery disease, %	13.7	14.2	11.8	0.001
Dialysis, %	1.8	2.1	0.7	<0.001
Cardiogenic shock, %	0.6	0.7	0.3	0.009
Emergent procedure, %	15.1	15.4	13.9	0.042
Troponin positive, %	32.8	31.9	36.9	<0.001
STEMI, %	12.4	12.4	12.5	0.922

PCI indicates percutaneous coronary intervention; CABG, coronary artery bypass grafting surgery; STEMI, ST‐segment myocardial infarction.

In the group who received PCI via a transfemoral approach, 177 (1.2%) patients had vascular access site complications. Access site hematomas occurred in 102 patients, bleeding requiring treatment with transfusion occurred in 69, and other vascular complications including retroperitoneal bleeding or pseudoaneurysm occurred in 50 patients. There were 9 patients with death from a vascular cause.

**Figure 1.**
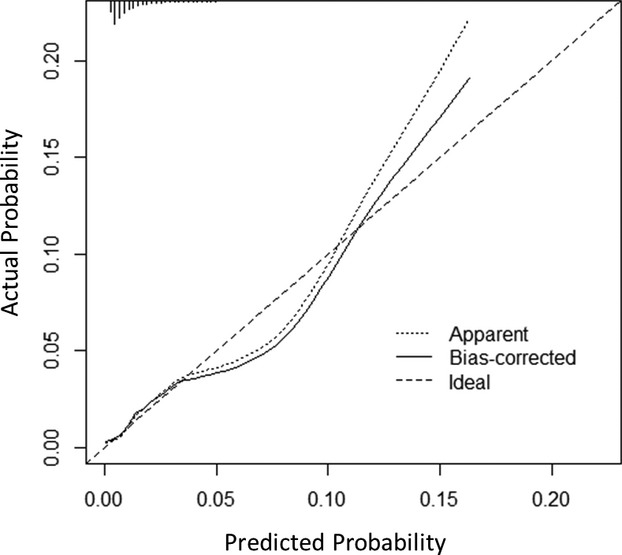
Observed vs predicted rates of vascular complications. This figure depicts observed (*y*‐axis) vs predicted (*x*‐axis) vascular access site complications in patients undergoing percutaneous coronary intervention (PCI) via transfemoral access. The bias‐corrected line represents the adjusted calibration curve accounting for optimism, as assessed by bootstrap validation. Differences in observed and predicted rates of events were small across all levels of risk (Hosmer–Lemeshow *P*=0.42).

The final model predicting vascular access site complications in those who had transfemoral arterial access had 8 significant predictors including elevated age, female gender, elevated troponin, chronic kidney disease, emergent procedure, prior PCI, diabetes, and peripheral artery disease (Table 2). The unadjusted c‐statistic of the model was 0.76, and was 0.72 after adjusting for optimism, demonstrating moderate discrimination for the model. After bootstrap resampling, a plot of observed versus predicted rates of vascular complications suggested good model calibration (calibration slope=0.92, Hosmer–Lemeshow goodness of fit=0.42). (Figure 1).

**Table 2.** Final Model for Vascular Complications in Patients Who Had Transfemoral Arterial Access

Variable	β	OR	Calibrated β	Calibrated OR	95% CI for Calibrated OR
Age (per 10 years)	0.262	1.300	0.241	1.272	1.119 to 1.449
Female (y/n)	0.980	2.664	0.902	2.464	1.845 to 3.289
Troponin elevated (y/n)	0.713	2.041	0.656	1.927	2.509 to 2.584
Chronic kidney disease					
GFR ≥60 mL min^−1^ 1.73 m^2^	Ref	Ref	Ref	Ref	Ref
GFR <60 mL min^−1^ 1.73 m^2^	0.245	1.278	0.225	1.252	0.914 to 1.718
Dialysis (y/n)	1.137	3.120	1.046	2.846	1.537 to 4.985
Emergent case (y/n)	0.478	1.612	0.439	1.551	1.100 to 2.188
Prior PCI (y/n)	−0.495	0.610	−0.455	0.634	0.460 to 0.874
PAD (y/n)	0.549	1.732	0.505	1.657	1.187 to 2.316
Diabetes (y/n)	0.430	1.537	0.396	1.486	1.110 to 1.985
Constant	−7.26		−6.679		

OR indicates odds ratio; CI, confidence interval; GFR, glomerular filtration rate; Ref, reference; PCI, percutaneous coronary intervention; PAD, peripheral artery disease.

**Figure 2.**
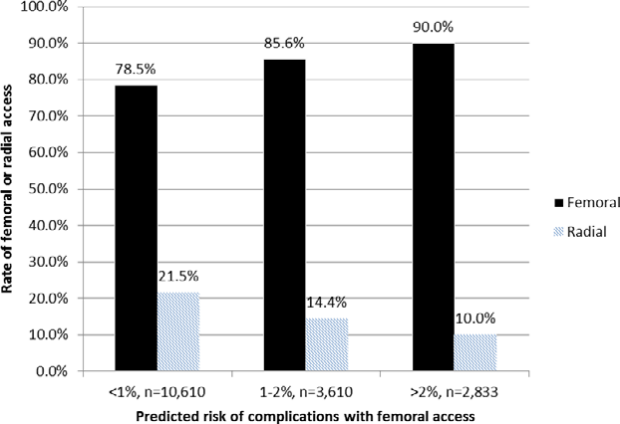
Rate of femoral or radial access according to predicted risk of transfemoral vascular access complications. Patients with higher predicted risk of complications via the transfemoral approach were less likely to receive a transradial approach (*P*<0.001).

**Figure 3.**
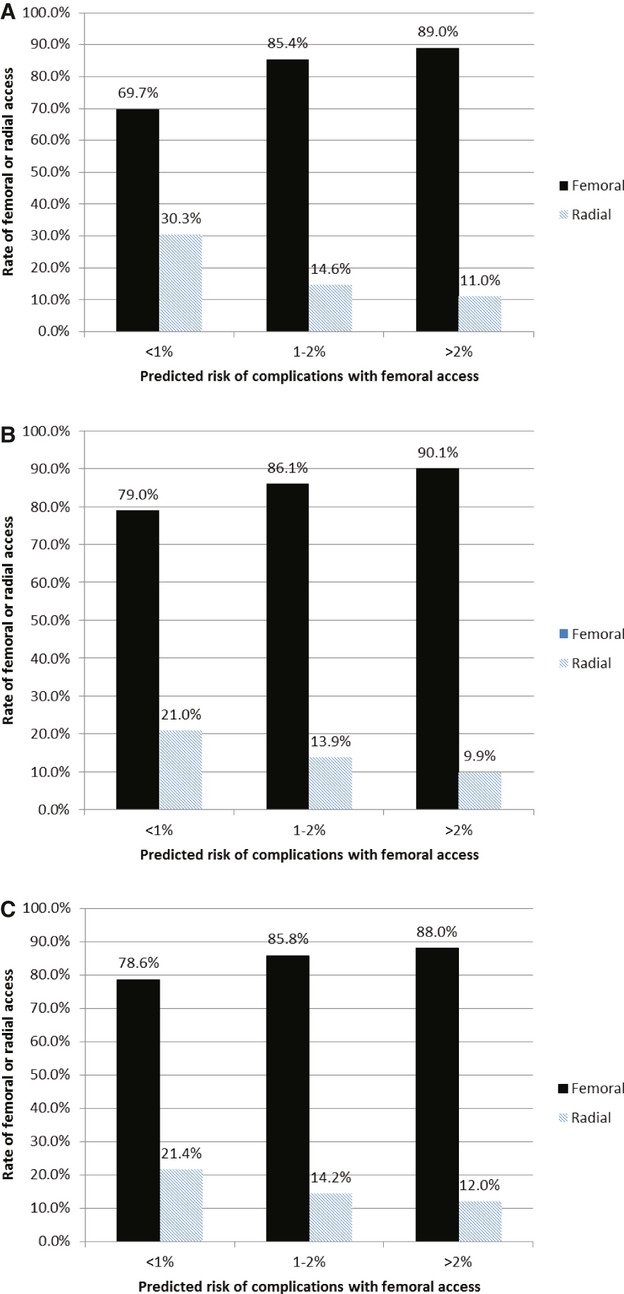
Rate of femoral or radial access according to predicted risk of transfemoral vascular access complications, stratified by presentation with ST‐segment myocardial infarction (STEMI). A, Patients presenting with STEMI (n=2171). B, Patients presenting without STEMI (n=14 386). C, Full study population excluding patients receiving dialysis treatment (n=16 747).

After application of the model predicting femoral vascular complications to all patients, including those who received transradial arterial access, patients with the highest predicted risk of complications via transfemoral arterial access were *less* likely to receive transradial access (*P*<0.001) (Figure 2). Similar results were seen in patients presenting for PCI with STEMI (*P*<0.001) and in those presenting without STEMI (*P*<0.001) (Figure 3). Similar results were also seen when the model was applied to the full study population excluding patients receiving dialysis treatment, for whom transradial access might have been limited due to the presence of arteriovenous fistulas or the desire to preserve future hemodialysis access sites (*P*<0.001) (Figure 3).

To quantify the relationship between the risk of transfemoral access site complications and the receipt of transradial arterial access, we generated logistic regression models. The odds ratio associated with the receipt of transradial access was 0.86 (95% CI, 0.82 to 0.90, *P*<0.0001) for each 1% increase in risk of transfemoral access site complications. Similarly, after adjusting for individual physician operators, the odds ratio associated with the receipt of transradial access was 0.83 (95% CI, 0.77 to 0.87, *P*<0.0001) for each 1% increase in risk of transfemoral access site complications.

## Discussion

We developed a model for vascular access site complications in patients undergoing PCI via a transfemoral arterial approach in a contemporary registry from 5 hospitals in Massachusetts. This model accounts for access site complications beyond bleeding alone, and therefore better reflects the true range of possible complications associated with PCI‐related arterial access. The model also ignores bleeding complications physically isolated from the arterial access site, such as gastrointestinal bleeding, likely to be modulated by the pharmacologic agents used around the time of PCI instead of the chosen arterial access site.

The model identified 8 independent factors associated with the development of vascular access site complications including: elevated age, female gender, elevated troponin, chronic kidney disease, whether a procedure was emergent, prior PCI, diabetes, and peripheral artery disease. Several of these factors, including elevated age, female gender, peripheral vascular disease, and whether a procedure was emergent have been previously identified as being associated with elevated procedural risk.^8,22^ Prior PCI has also previously been shown to be associated with less risk of post‐PCI bleeding, similar to our findings.^22^

The risk algorithm presented here allows the calculation of a specific risk of access site complications for a given patient and makes explicit the contribution of each risk factor. We believe that this model is highly actionable in clinical practice because we intentionally chose to model only clinical variables that would be available before the PCI, or even routine coronary angiography, was performed. A prior risk model predicting vascular access complications relies on knowledge of the coronary anatomy as well as knowledge of medication use that is often initiated during or around the time of PCI.^8^ We have also intentionally excluded other factors that are defined at the time of the procedure, or are procedural‐related factors themselves. The femoral arterial anatomy, the site of a femoral arteriotomy, and the decision to use vascular closure devices are either defined at the time of angiography or during the PCI and thus are not useful for preprocedural prediction.

The rate of transradial arterial access in this study population was higher (17.8%) than the published rates across the United States (<1.5%).^17^ When applying the model that we developed for transfemoral arterial access site complications to the entire population of PCI patients, including those who received transradial access, we intended to determine if clinicians in routine practice were employing a strategy for the use of transradial arterial access that would be expected to prevent the highest number of complications. Instead, we found that the individuals at highest risk for access site complications via a transfemoral approach were the least likely to receive transradial access during PCI. This pattern of use, known as the risk‐treatment paradox, has been described previously for other therapies including statin treatment in the elderly,^28^ anticoagulation for atrial fibrillation,^29^ and the use of bleeding avoidance strategies, such as anticoagulation with bivalirudin, during PCI,^30^ and may represent an important area for quality improvement.

Our observation led to us to explore several explanatory hypotheses. First, we hypothesized that a desire to perform the procedure quickly, particularly in patients presenting with STEMI, led to increased rates of transfemoral access. In a previous meta‐analysis comparing clinical outcomes in patients who undergo PCI with transradial compared with transfemoral arterial access, procedure times were longer in the transradial group.^14^ The data presented in our analysis do not support this hypothesis as the sole explanation of our findings, however. We demonstrate that in those presenting with both STEMI and those presenting for PCI without STEMI (including both patients with stable coronary artery disease and those with non‐ST‐segment elevation acute coronary syndromes), patients at highest risk for access site complications via a transfemoral approach were most likely to receive transfemoral access in each subgroup. We also performed an analysis excluding patients receiving dialysis treatment as these patients are often not candidates for transradial arterial access due to the desire to preserve possible hemodialysis access points for the future. After excluding this population, patients at highest risk for access site complications via a transfemoral approach were still most likely to receive transfemoral access.

There are several other factors that may be driving our findings. It is possible that the factors associated with access site complications are themselves associated with increased procedural complexity and treating physicians prefer to perform complex cases (either due to patient presentation, comorbidities, or anatomic factors) via a transfemoral approach. We also hypothesize that these data reflect the learning curve of physicians who are increasing the use of transradial PCI over time, but who begin with simpler cases while gaining experience. These data cannot adequately address these hypotheses.

While an increase in the use of transradial approaches in patients at high risk for femoral access complications would likely reduce such complications, the choice of access site should be weighed alongside other clinical considerations. Weighing the technical success of the procedure, and minimizing risk of nonaccess site adverse events such as reinfarction or future stent thrombosis, may be more important than small added risk of vascular access site complications for certain patients. However, periprocedural bleeding during PCI has been shown to be associated with recurrent adverse cardiac events and elevated rates of all‐cause mortality.^9,31–34^ Furthermore, the chief benefit of radial access in randomized trials has been the prevention of access site complications.^13^ Thus, since one of the primary purposes of choosing radial arterial access compared with femoral arterial access for PCI is to prevent vascular access site complications, including bleeding, clinicians would benefit from the tools to make rational decisions in this regard.

We acknowledge, however, that increasing the use of transradial PCI is also not exactly akin to increasing the use of evidence‐based medication therapies, for instance. Technical expertise and proficiency are required before transradial PCI can be safely performed, particularly in technically challenging cases. Sites need to move from a stage early in the transradial learning curve where operators perform the simplest cases via a transradial approach to a “radial‐first” stage where transradial access is the preferred method of arterial access, as has been suggested by the recent European Society of Cardiology Consensus Statement regarding transradial PCI.^35^ From our analyses, it is difficult to determine where these 5 institutions are on the learning curve for transradial PCI. Different research methodologies will be needed to adequately address this question in the future.

There are several other important limitations in these analyses. Our study was observational in nature, and thus direct comparisons of complication rates among transfemoral and transradial PCI procedures may be subject to residual confounding. Next, although our study describes a multicenter experience including more than 17 000 patients undergoing PCI in clinical practice, it was limited to 5 hospitals in Massachusetts where transradial access utilization is higher than the reported national average. In addition, while our model was internally validated via bootstrapping, incorporates risk factors that have been previously described as being associated with procedural complications, and compares favorably in terms of discrimination and calibration of other PCI prediction models,^22,36–37^ the study remains to be validated in an external dataset. As such, our findings may not be generalizable to all populations. Next, the clinical endpoints in this study were not adjudicated independently, potentially leading to underreporting particularly of less significant access site complications. Also, other factors that might influence vascular access site complications were not considered in the models due to our desire to use preprocedural factors only. Thus, some factors that influence bleeding, such as anticoagulant and antiplatelet agents as well as vascular closure devices, were not accounted for in the models. Finally, longterm outcomes are unavailable for this population so we do not know the influence of vascular access site complications on a patient's clinical status after hospital discharge.

## Conclusions

We developed and internally validated a model predicting risk for the development of vascular access site complications during PCI via a transfemoral approach. We then demonstrated that among all patients who undergo PCI, those at lowest risk for access site complications were most likely to receive transradial arterial access while those at highest risk for vascular access site complications were the most likely to undergo transfemoral arterial access. If transradial arterial access is to be employed rationally as a strategy to avoid vascular access site complications, there is significant room for improvement in its use.
